# An Expeditious and Greener Synthesis of 2-Aminoimidazoles in Deep Eutectic Solvents

**DOI:** 10.3390/molecules21070924

**Published:** 2016-07-16

**Authors:** Martina Capua, Serena Perrone, Filippo Maria Perna, Paola Vitale, Luigino Troisi, Antonio Salomone, Vito Capriati

**Affiliations:** 1 Dipartimento di Scienze e Tecnologie Biologiche ed Ambientali, Università del Salento, Prov.le Lecce-Monteroni, Lecce I-73100, Italy; martina.capua@unisalento.it (M.C.); serena.perrone@unisalento.it (S.P.); luigino.troisi@unisalento.it (L.T.); 2 Dipartimento di Farmacia-Scienze del Farmaco, Università degli Studi di Bari «Aldo Moro», Consorzio C.I.N.M.P.I.S., Via E. Orabona 4, Bari I-70125, Italy; filippo.perna@uniba.it (F.M.P.); paola.vitale@uniba.it (P.V.)

**Keywords:** 2-aminoimidazoles, deep eutectic solvents, green chemistry, heterocyclization, guanidines, α-chloroketones

## Abstract

A high-yield one-pot two-step synthesis of 2-aminoimidazoles (2-AI), exploiting an under-air heterocyclodehydration process between α-chloroketones and guanidine derivatives, and using deep eutectic solvents (DESs) as nonconventional, “green” and “innocent” reaction media, has been accomplished successfully. The combination of either glycerol or urea with choline chloride (ChCl) proved to be effective for decreasing the reaction time to about 4–6 h in contrast to the 10–12 h usually required for the same reaction run in toxic and volatile organic solvents and under an argon atmosphere. In addition, the use of the ChCl–urea as a DES also enables the direct isolation of triaryl-substituted 2-AI derivatives by means of a simple work-up procedure consisting in filtration and crystallization, and allows the recycle of the DES mixture. A plausible mechanism highlighting the potential role played by hydrogen bonding catalysis has also been illustrated.

## 1. Introduction

**Figure 1:**
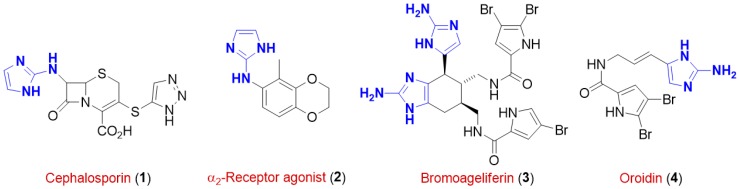
Structural formulas of some pharmacologically-relevant 2-aminoimidazoles.

The American Chemical Society′s (ACS) Green Chemistry Institute Pharmaceutical Roundtable (GCIPR), founded in 2005 with the aim of catalyzing the integration of green chemistry and engineering into the pharmaceutical industry, strives for the need to replace conventional hazardous volatile organic solvents (VOCs) in favor of safe, green and biorenewable reaction media that are not based on crude petroleum. Solvents are, indeed, still responsible for most waste generated in the chemical industries and laboratories as they account for 80%–90% of mass utilization in a typical pharmaceutical/fine chemical operational process [1]. Thus, there is a global and increasing demand for the development of renewable solvents not based on crude petroleum [2]. 2-Aminoimidazole (2-AI) derivatives are an extremely important class of substituted nitrogen-containing heterocycles whose core has been recognized as a key and essential structural element in a wide range of bioactive molecules such as the antibiotic **1** belonging to the family of cephalosporins [3], the α_2_-receptor agonist **2** [4], and the naturally-occurring sponge metabolites bromoageliferin **3** and oroidin **4**, which are known for their anti-biofilm activity [5,6] (Figure 1). In addition, they have found wide application in coordination chemistry [7,8], in organocatalysis [9], and proven to be either valuable pharmacophores in medicinal chemistry for the development of high value-added molecules for discovery based research [10] or useful building blocks for the design of modulators of various small molecule drugs being bioisosteres of guanidine, benzamidine and triazole rings [11].

As such, novel synthetic approaches for the construction of 2-AI scaffolds are continuously proposed. Classical methods rely upon condensation reactions, decoration of imidazole derivatives and heterocyclic exchange reactions [10,11,12,13]. More recently, various metal-mediated cycloguanylation reactions have also been proposed [14,15,16,17,18,19]. Despite these synthetic efforts, however, the construction of 2-AIs is still performed in toxic, hazardous and expensive VOCs (e.g., THF, DMF, toluene, etc.) and reactions en route to these heterocycles are generally carried out under an inert atmosphere with strict exclusion of humidity, thereby adopting protocols of limited utility for industry users. Deep eutectic solvents (DESs) represent an emerging class of unconventional solvents that have become of growing interest both at academic and industrial levels. Compared to conventional VOCs, DESs show high thermal stability, non-flammability and practically no vapor pressure, therefore low volatility. Formation of DESs can be easily achieved by simply mixing together and gently warming at least two safe, cheap, renewable and biodegradable components, generally a hydrogen bond donor and a hydrogen bond acceptor which are capable of forming a eutectic mixture having a melting point lower than either of the individual components. Typical DES components (e.g., choline chloride (ChCl), urea, glycerol (Gly), natural carboxylic acids, amino acids and carbohydrates, polyalcohols, etc.) come from renewable sources; thus, their biodegradability is extraordinarily high, and their toxicity is non-existent or very low [20,21,22,23,24,25]. The major research effort to date has been mainly concentrated on replacement solvents for metal finishing applications [24], for biomass valorization [23], for extraction processes [26], and in polymerizations and materials science [27,28]. Emerging applications are in the fields of organometallic chemistry [29,30,31,32,33], metal-catalyzed reactions [34,35,36], and organocatalysis [37,38,39]. In this manuscript, we describe the first one-pot two-step synthesis of 2-AIs exploiting an alkylation reaction followed by a cyclocondensation process between α-chloroketones and guanidine derivatives successfully run in eutectic mixtures as “innocent” and biorenewable DES media. The whole transformation has been proven to proceed in high yield within short reaction times (4 to 6 h) compared to conventional VOCs (10 to 12 h), and could be successfully carried out under air.

## 2. Results and Discussion

Intrigued by the potential to synthesize substituted 2-AIs employing DESs as effective and “green” reaction media, we began our study by re-investigating the one-pot synthesis of 2-AIs published by Webber in the 1990s [12] but using the aforementioned unconventional solvents. In this seminal paper, the cyclization reaction between α-haloketones and an excess of *N*-acetylguanidine (at least three equivalents) proved to be effective upon stirring the reaction mixture in anhydrous DMF, under argon and at room temperature for four days, or at reflux in CH_3_CN for 16 h. The solvent polarity was also studied, and inferior results were obtained when the reaction was run in CHCl_3_, DME, or DMSO. A type III DES [21], namely ChCl–Gly (1:2), was selected as a model eutectic mixture for studying such heterocyclodehydration reaction. ChCl, also known as vitamin B4, is one of the most widely used ammonium salts in forming DESs. It is a very cheap (ca. 2 €·kg^−1^) and biodegradable material, and is produced on the scale of a million metric tons per year as an additive in chicken food for accelerating growth. When a solution of α-chloroketone **1a** (1.0 mmol), substituted guanidine **2a** (1.3 mmol) and Et_3_N (1.0 mmol) was vigorously stirred at 80 °C and under air in the above DES mixture, the complete disappearance of the starting ketone took place after ca. 4 h, as revealed by GC-MS analysis. Because of the high solubility of the above DES mixture in water, a very simple reaction workup was required: the desired AI **3a** could indeed be isolated in 85% yield by simple dilution with equal volume mixture of water and AcOEt, followed by separation of the organic layer and removal of the solvent under reduced pressure (Table 1, entry 1) [40].

On the other hand, when performed in anhydrous THF and under an argon atmosphere, such condensation process required a longer reaction time (ca. 12 h) and the use of reflux conditions to go to completion. After partitioning the crude between AcOEt and water, product **3a** could be finally isolated in 81% yield after column chromatography on silica gel (Table 1, entry 2). Further experimentation to explore the scope of the reaction established that assorted aliphatic (**1b**,**c**) and aromatic α-chloroketones with electron-donating (**1d**,**e**), electron-withdrawing (**1f**) and fluorine (**1g**) substituents, all proved to be competent partners straightforwardly providing the expected 2-AI derivatives **3b**–**g** in very good yields (70%–83%) (Table 1, entries 3, 5, 7, 9, 11, and 13). The corresponding reactions, run in anhydrous THF and under an argon atmosphere, led to the desired products in comparable yields (71%–85%), albeit after 10–12 h reflux (Table 1, entries 4, 6, 8, 10, 12, and 14). With the aim to extending the scope of the reaction even more, it was interesting to observe that such condensation processes worked well in a relatively short reaction time (4–6 h) also by reacting α-chloroketones **1a** and **1b** with a different guanidine derivative (**2b**) in the above ChCl–Gly (1:2) DES mixture, thereby affording the corresponding 2-AIs **3h** and **3i** in 73% and 72% yields, respectively, (Table 1, entries 15 and 17). As for VOCs, similar yields (70%–76%) were obtained using a polar solvent such as EtOH, again to the detriment of the reaction time (12 h reflux) (Table 1, entries 16 and 18).

We were also keen to explore for this transformation another common and widely used DES, namely the ChCl–urea (1:2) eutectic mixture. At the outset of this investigation, we were rather skeptical about the successful outcome of the reaction. In fact, it has been recently reported by Shankarling and co-workers that α-bromoketones are able to react with the urea component of a DES mixture to produce 2-aminooxazole derivatives as the final products [41]. In this case, a DES is a “non-innocent” medium as it becomes an integral part of the starting materials. Remarkably, upon mixing α-chloroketone **1g** with guanidine derivative **2a**, and heating the reaction mixture to up to 80 °C, 2-AI **3g** was found to precipitate directly from the above DES mixture during the reaction, and could be isolated by simple filtration on paper, thereby avoiding the typical organic solvent extraction procedure performed at the end of the reaction. An ^1^H-NMR analysis of the product **3g** showed a purity of about 95%, which could be further increased to >98% after crystallization from Et_2_O by using hexane as a non solvent, the Et_2_O/hexane ratio being almost 8/2 (yield of **3g**: 85%; Table 2, entry 6).

Overall, the reaction proved to be extraordinarily clean (no other by-products could be detected) and more user-friendly compared to the procedure using ChCl–Gly (1:2) as the eutectic mixture. In addition, the ChCl–urea DES could be recovered and recycled. Indeed, after filtration of **3g**, the addition of 2 mL of water to the DES (reaction performed on 1.0 mmol scale of substrate; see Table 2) allowed the unreacted guanidine together with small quantities of other organic byproducts to precipitate and to be removed by filtration. Subsequent removal of water from the aqueous layer under vacuum furnished a DES mixture that could be successfully reused for an additional run without significant loss of **3g** yield (80%). However, starting from the third cycle, a drop in the chemical yield was noticed: 60%. We have then explored the reactivity of other aromatic and aliphatic α-chloroketones **1a**,**b**,**d**–**f** toward guanidines **2a**,**b** in the above ChCl–urea medium. We were pleased to find that, again, all the planned condensation reactions proved to be clean and effective providing the desired 2-AIs **3a**,**b**,**d**–**f**,**h** in good yields (65%–88%; Table 2, entries 1–5 and 7). It is worth noting that, similar to what has been shown for compound **3g**, triaryl-substituted imidazoles **3a**,**d**–**f** could also be successfully isolated with a purity >98% (^1^H-NMR) directly from the reaction mixture by simple filtration followed by crystallization (Et_2_O/hexane). Conversely, the isolation of the diaryl-substituted imidazoles 2-AIs **3b**,**h** required a work-up procedure followed by a column chromatography on silica gel (See Materials and Methods).

An interplay of factors may be responsible of the results obtained from the use of the ChCl–urea eutectic mixture: the higher nucleophilicity of guanidine **2a** compared to that of urea (which does not compete despite being the bulk medium), the stronger intermolecular hydrogen-bonding interactions urea establishes with ChCl compared to Gly [42], which may reduce even more the solubility of the final product in such a DES mixture.

**Scheme 1:**
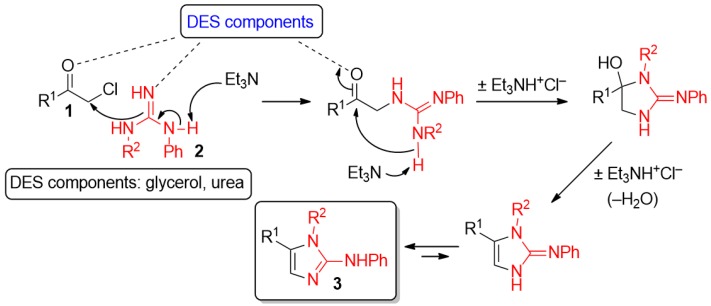
Plausible mechanism for the formation of 2-aminoimidazoles in DES mixtures.

Hydrogen bond catalysis promoted by DESs components (e.g., glycerol and urea) may contribute as well to activate both carbonyl and guanidine groups, thereby exalting their electrophilic and nucleophilic character, respectively. This offers a possible explanation for the increase of reaction rate observed for such heterocyclodehydration processes in DES media compared to the longer reaction times needed in VOCs, instead. The role of a DES as a catalytic active species has also been suggested for other reactions [41,43,44,45,46,47,48,49]. A plausible and simple mechanism of formation of 2-AIs **3** from α-chloroketones **1** and guanidines **2** is depicted in Scheme 1. The tertiary base Et_3_N may be playing an active role in triggering as well as in driving the reaction to completion as in its absence the reaction proceeds quite sluggishly [50].

## 3. Materials and Methods

### 3.1. General Informations

Reagents and solvents, unless otherwise specified, were purchased from Sigma-Aldrich (Sigma-Aldrich, St. Louis, MO, USA) and used without any further purification. THF was purified by distillation from sodium/benzophenone before use. Petroleum ether refers to the 40–60 °C boiling fraction. The ^1^H- and the ^13^C-NMR spectra were recorded on a Bruker spectrometer (Bruker, Billerica, MA, USA) operating at 400.13 MHz for ^1^H and 100.62 MHz for ^13^C, with CDCl_3_ as the solvent and TMS as an internal standard (δ = 7.26 ppm for ^1^H spectra; δ = 77.0 ppm for ^13^C spectra). The IR spectra were recorded on a Jasco 4100 FT-IR spectrometer (Jasco, Easton, MD, USA) as CHCl_3_ solution. GC-MS analyses were performed with a gas-chromatograph equipped with a 5% phenylpolymethylsiloxane capillary column, 30 m, 0.25 mm i.d., and a mass-selective detector operating at 70 eV. The electrospray ionization (HRMS (ESI)) experiments were carried out in a hybrid Q-TOF mass spectrometer (Agilent, Santa Clara, CA, USA) equipped with an ion-spray ionization source. MS (+) spectra were acquired by direct infusion (5 mL∙min^−1^) of a solution containing the appropriate sample (10 pmol∙mL^−1^) dissolved in a solution 0.1% acetic acid, MeOH/H_2_O (50:50) at the optimum ion voltage of 4800 V. The nitrogen gas flow was set at 30 psi (pounds per square inch) and the potentials of the orifice, the focusing ring and the skimmer were kept at 30, 50 and 25 V relative to ground, respectively. TLC was performed on silica gel plates with F-254 indicator (Merck, Darmstadt, Germany); viewing was by UV light (254 nm) or phosphomolybdic acid staining solution. Chromatographic separations were performed on silica gel (63–200 mesh) using petroleum ether/AcOEt mixture as the eluent. All reactions involving air-sensitive reagents were performed under an atmosphere of nitrogen in oven-dried glassware by using syringe/septum cap techniques. The deep eutectic solvents ChCl–Gly (1:2 mol∙mol^−1^) and ChCl–urea (1:2 mol∙mol^−1^) were prepared by gently heating under stirring at 70 °C for 5 min the corresponding individual components until a clear solution was obtained. Reagent **2b** was prepared as guanidinium carbonate **2b** H_2_CO_3_ according to literature procedure [51]. For the preparation of compounds **3a**–**g** in THF and **3h**,**i** in EtOH, see Supplementary Materials.

### 3.2. Synthesis of 2-Aminoimidazoles ***3a**–**g*** in the DES ChCl–Gly (1:2) (Table 1)

The appropriate α-chloroketone **1** (1.0 mmol), guanidine **2a** (1.3 mmol) and Et_3_N (1 mmol) were added to the ChCl–Gly eutectic mixture (2 g) under magnetic stirring, and the mixture was then heated to 80 °C for a period of 4–6 h, until the ketone **1** disappeared, as revealed by GC-MS analysis. After this time, the mixture was cooled to room temperature and 5 mL of H_2_O were added. The resulting aqueous suspension was then extracted with AcOEt (3 × 10 mL). The combined organic phases were dried over Na_2_SO_4_ and concentrated in vacuo. The crude product was purified by flash-chromatography (silica gel; petroleum ether/AcOEt 80:20–95:5) to give the desired aminoimidazoles **3a**–**g**.

### 3.3. Synthesis of 2-Aminoimidazoles ***3h**,**i*** in the DES ChCl–Gly (1:2) (Table 1)

Guanidinium carbonate **2b** H_2_CO_3_ (1.3 mmol) and KOH (1.3 mmol) were added to the ChCl–Gly eutectic mixture (2 g) under magnetic stirring, and the mixture was then heated to 80 °C for a period of 30 min, so as to liberate the free base of guanidine **2b** in situ. After this time, α-chloroketone **1** and Et_3_N (1.3 mmol) were added, and the reaction stirred at 80 °C for 4–6 h until the ketone **1** disappeared, as revealed by GC-MS analysis. The reaction mixture was then cooled to room temperature and 5 mL of H_2_O were added. The resulting aqueous suspension was extracted with AcOEt (3 × 10 mL) and the combined organic phases were dried over Na_2_SO_4_ and concentrated in vacuo. The crude product was purified by flash-chromatography (silica gel; petroleum ether/AcOEt 20:80–40:60) to give the desired aminoimidazoles **3h**,**i**.

### 3.4. Synthesis of 2-Aminoimidazoles ***3a**,**b**,**d**–**g*** in the DES ChCl–Urea (1:2) (Table 2)

The appropriate α-chloroketone (**1a**,**b**,**d**–**g**) (1.0 mmol), guanidine **2a** (1.3 mmol) and Et_3_N (1 mmol) were added to the ChCl–urea eutectic mixture (2 g) under magnetic stirring, and the mixture was then heated to 80 °C for a period of 4–6 h until the ketone **1** disappeared, as revealed by GC-MS analysis. After this time, the reaction mixture was cooled to room temperature and the purification of product **3a**,**d**–**g** was achieved by filtration on paper. The brown solid obtained was further purified by crystallization from Et_2_O/hexane (ca. 8/2 ratio). The purification of compounds **3b** was achieved as following: after the completion of the reaction, the mixture was cooled to room temperature and 5 mL of H_2_O were added. The resulting aqueous suspension was extracted with AcOEt (3 × 10 mL) and the combined organic phases were dried over Na_2_SO_4_, and concentrated in vacuo. The crude product was purified by flash-chromatography (silica gel; petroleum ether/AcOEt 20:80–40:60) to give the desired aminoimidazoles **3b**.

### 3.5. Synthesis of 2-Aminoimidazoles ***3h*** in the DES ChCl–Urea (1:2) (Table 2)

Guanidinium carbonate **2b** H_2_CO_3_ (1.3 mmol) and KOH (1.3 mmol) were added to the ChCl–urea eutectic mixture (2 g) under magnetic stirring, and the mixture was then heated to 80 °C for a period of 30 min, so as to liberate the free base of guanidine **2b** in situ. After this time, α-chloroketone **1a** and Et_3_N (1.3 mmol) were added and the reaction stirred at 80 °C for 4 h until the ketone **1a** disappeared, as revealed by GC-MS analysis. The reaction mixture was then cooled to room temperature and 5 mL of H_2_O were added. The resulting aqueous suspension was extracted with AcOEt (3 × 10 mL) and the combined organic phases were dried over Na_2_SO_4_ and concentrated in vacuo. The crude product was purified by flash-chromatography (silica gel; petroleum ether/AcOEt 20:80–40:60) to give the desired aminoimidazole **3h**.

### 3.6. Characterization Data of Compounds ***3a**–**i*** (Table 1)

*N,1,5-Triphenyl-1H-imidazol-2-amine* (**3a**). Pale yellow solid (264 mg, 85%), m.p. 116–118 °C. ^1^H-NMR (400.13 MHz, CDCl_3_): δ 6.10 (1 H, broad s, NH, exchanges with D_2_O), 6.86–6.90 (1 H, m, Ph), 7.09 (1 H, s, imidazole H), 7.22–7.46 (12 H, m, Ph), 7.83 (2 H, d, *J* = 7.3 Hz, Ph) ppm. ^13^C-NMR (100.62 MHz, CDCl_3_): δ 112.0, 116.2, 120.8, 124.5, 125.1, 126.5, 128.2, 128.4, 128.9, 129.9, 134.0, 136.2, 138.2, 141.1, 143.3 ppm. FT-IR (CHCl_3_): ν 3410 (NH), 3011, 2963, 2933, 2842, 1735, 1656, 1601, 1503, 1393 cm^−1^. GC/MS (70 eV): *m*/*z* (%) 311 (100) [M]^+^, 310 (35), 295 (33), 207 (49), 104 (20). HRMS (ESI): calcd. for C_21_H_18_N_3_ [M + H]^+^ 312.1501; found 312.1503.

*5-tert-Butyl-N,1-diphenyl-1H-imidazol-2-amine* (**3b**). Yellow oil (215 mg, 74%). ^1^H-NMR (400.13 MHz, CDCl_3_): δ 1.34 [9 H, s, (CH_3_)_3_C], 5.96 (1 H, broad s, NH, exchanges with D_2_O), 6.57 (1 H, s, imidazole H), 6.79–6.89 (1 H, m, Ph), 7.16–7.20 (4 H, m, Ph), 7.35–7.45 (5 H, m, Ph) ppm. ^13^C-NMR (100.62 MHz, CDCl_3_): δ 29.8, 31.8, 110.1, 115.7, 120.3, 124.8, 127.6, 128.9, 129.7, 137.0, 141.8, 142.2, 149.1 ppm. FT-IR (CHCl_3_): ν 3424 (NH), 3030, 2967, 2870, 1725, 1676, 1545, 1479, 1419, 1316 cm^−1^. GC/MS (70 eV): *m*/*z* (%) 291 (70) [M]^+^, 276 (100), 104 (15),77 (32). HRMS (ESI): calcd. for C_19_H_22_N_3_ [M + H]^+^ 292.1814; found 292.1813.

*5-Methyl-N,1-diphenyl-1H-imidazol-2-amine* (**3c**). Yellow oil (174 mg, 70%). ^1^H-NMR (400.13 MHz, CDCl_3_): δ 2.25 (3 H, s, CH_3_), 5.94 (1 H, broad s, NH, exchange with D_2_O), 6.58 (1 H, s, imidazole H), 6.86–6.99 (1 H, m, Ph), 7.20–7.49 (9 H, m, Ph) ppm. ^13^C-NMR (100.62 MHz, CDCl_3_): δ 13.9, 112.8, 116.2, 120.7, 125.0, 127.8, 129.0, 129.8, 134.9, 136.7, 141.6. 142.5 ppm. FT-IR (CHCl_3_): ν 3424 (NH), 3032, 2967, 2905, 2871, 1725, 1676, 1543, 1480, 1419, 1316 cm^−1^. GC/MS (70 eV): *m*/*z* (%) 249 (100) [M]^+^, 248 (68), 234 (40), 104 (25), 77 (38). HRMS (ESI): calcd. for C_16_H_16_N_3_ [M + H]^+^ 250.1345; found 250.1348.

*N,1-Diphenyl-5-p-tolyl-1H-imidazol-2-amine* (**3d**). Yellow solid (260 mg, 80%), m.p. 120–122 °C. ^1^H-NMR (400.13 MHz, CDCl_3_): δ 2.33 (3 H, s, CH_3_), 6.10 (1 H, broad s, NH, exchanges with D_2_O), 6.84–6.87 (1 H, s, Ph), 7.04 (1 H, s, imidazole H), 7.15–7.21 (4 H, m, Ar), 7.32–7.43 (7 H, m, Ar), 7.72 (2 H, d, *J* = 7.3 Hz, Ar) ppm. ^13^C-NMR (100.62 MHz, CDCl_3_): δ 21.1, 111.5, 116.1, 120.6, 124.4, 125.0, 128.0, 128.8, 129.0, 129.8, 131.2, 136.0, 136.3, 138.3, 141.2, 143.1 ppm. FT-IR (CHCl_3_): ν 3409 (NH), 3011, 2963, 2930, 2845, 1736, 1656, 1602, 1503, 1391 cm^−1^. GC/MS (70 eV): *m*/*z* (%) 325 (100) [M]^+^, 324 (26), 207 (50), 104 (12), 77 (23). HRMS (ESI): calcd. for C_22_H_20_N_3_ [M + H]^+^ 326.1658; found 326.1655.

*5-(4-Methoxyphenyl)-N,1-diphenyl-1H-imidazol-2-amine* (**3e**). Dark yellow solid (269 mg, 79%), m.p. 139–141 °C. ^1^H-NMR (400.13 MHz, CDCl_3_): δ 3.83 (3 H, s, OCH_3_), 6.11 (1 H, broad s, NH, exchanges with D_2_O), 6.87–6.94 (3 H, m, Ar), 7.07 (1 H, s, imidazole H), 7.23–7.52 (9 H, m, Ar), 7.76 (2 H, d, *J* = 7.4 Hz, Ar) ppm. ^13^C-NMR (100.62 MHz, CDCl_3_): *δ* = 55.2, 111.0, 113.9, 116.3, 120.8, 125.2, 125.8, 126.9, 128.2, 129.0, 130.0, 136.5, 138.2, 141.3, 143.2, 158.5 ppm. FT-IR (CHCl_3_): ν 3410 (NH), 3067, 3011, 2963, 1933, 2842, 1735, 1656, 1601, 1503, 1393 cm^−1^. GC/MS (70 eV): *m*/*z* (%) 341 (100) [M]^+^, 340 (20), 326 (15), 207 (48), 104 (12), 77 (25). HRMS (ESI): calcd. for C_22_H_20_N_3_O [M + H]^+^ 342.1607; found 342.1609.

*5-(4-Chlorophenyl)-N,1-diphenyl-1H-imidazol-2-amine* (**3f**). Yellow solid (276 mg, 80%), m.p. 106–108 °C. ^1^H-NMR (400.13 MHz, CDCl_3_): δ 6.06 (1 H, broad s, NH, exchanges with D_2_O) 6.92 (1 H, t, *J* = 7.3 Hz, Ar), 7.10 (1 H, s, imidazole H), 7.24–7.53 (11 H, m, Ar), 7.75 (2 H, d, *J* = 7.4 Hz, Ar) ppm. ^13^C-NMR (100.62 MHz, CDCl_3_): δ 112.2, 116.4, 121.1, 125.3, 125.8, 128.5(2C), 129.0, 130.1, 131.9, 132.6. 136.1, 137.3, 140.8, 143.5 ppm. FT-IR (CHCl_3_): ν 3425 (NH), 3067, 3010, 2929, 1682, 1598, 1545, 1497, 1484, 1451, 1385 cm^−1^. GC/MS (70 eV): *m*/*z* (%) 345 (100) [M]^+^, 344 (30), 329 (28), 207 (51), 104 (15), 77 (32). HRMS (ESI): calcd. for C_21_H_17_ClN_3_ [M + H]^+^ 346.1112; found 346.1109.

*5-(4-Fluorophenyl)-N,1-diphenyl-1H-imidazol-2-amine* (**3g**). Pale yellow solid (273 mg, 83%), m.p. 101–103 °C. ^1^H-NMR (400.13 MHz, CDCl_3_): δ 6.46 (1 H, broad s, NH, exchanges with D_2_O), 7.15 (1 H, t, *J* = 7.3 Hz, Ar), 7.26 (1 H, s, imidazole H),7.31 (2 H, t, *J* = 8.7 Hz, Ar), 7.49 (2 H, t, *J* = 7.9 Hz, Ar), 7.57–7.71 (7 H, m, Ar), 8.04–8.08 (2 H, m, Ar) ppm. ^13^C-NMR (100.62 MHz, CDCl_3_): δ 111.5, 115.0 (d, *J* = 21.3 Hz), 120.6, 124.9, 125.9, 125.9 (d, *J* = 5.3 Hz), 128.0, 128.7, 129.7, 130.2, 136.0, 137.2, 141.0, 143.2, 161.5 (d, *J* = 244.7 Hz) ppm. FT-IR (CHCl_3_): ν 3424 (NH), 3067, 3045, 3009, 2970, 1681, 1600, 1575, 1538, 1499, 1451, 1386, 1338, 1232 cm^−1^. GC/MS (70 eV): *m*/*z* (%) 329 (100) [M]^+^, 328 (29), 313 (29), 207 (35), 104 (15), 77 (25). HRMS (ESI): calcd. for C_21_H_17_FN_3_ [M + H]^+^ 330.1407; found 330.1405.

*N,5-Diphenyl-1H-imidazol-2-amine* (**3h**). Pale yellow solid (172 mg, 73%), m.p. 116–118 °C. ^1^H-NMR (400.13 MHz, CDCl_3_): δ 4.90 (2 H, broad s, 2 × NH, exchanges with D_2_O), 6.99 (1 H, s, imidazole H), 7.17–7.20 (1 H, m, Ph), 7.31–7.49 (7 H, m, Ph), 7.69 (2 H, d, *J* = 7.3 Hz, Ph) ppm. ^13^C-NMR (100.62 MHz, CDCl_3_): δ 111.2, 124.3, 124.5, 126.3, 127.7, 128.4, 129.8, 134.1, 136.9, 137.7, 148.0 ppm. FT-IR (CHCl_3_): ν 3464, 3067, 3037, 3009, 2962, 2930, 2855, 1616, 1600, 1504, 1454, 1354 cm^−1^. GC/MS (70 eV): *m*/*z* (%) 235 (100) [M]^+^, 234 (15), 193(12), 165 (15), 132 (75), 131 (35), 104 (30), 77 (25). HRMS (ESI): calcd. for C_15_H_14_N_3_ [M + H]^+^ 236.1188; found 236.1186.

*5-tert-Butyl-N-phenyl-1H-imidazol-2-amine* (**3i**). Pale yellow oil (155 mg, 72%). ^1^H-NMR (400.13 MHz, CDCl_3_): δ 1.27 (9 H, s, (CH_3_)_3_C), 4.40 (2 H, broad s, 2 × NH, exchanges with D_2_O), 6.38 (1 H, s, imidazole H),7.13–7.18 (2 H, m, Ph), 7.32–7.35 (1 H, m, Ph), 7.44–7.47 (2 H, m, Ph) ppm. ^13^C-NMR (100.62 MHz, CDCl_3_): δ 29.8, 31.4, 108.8, 124.3, 125.2, 127.2, 128.9, 137.8, 148.1 ppm. FT-IR (CHCl_3_): ν 3422 (NH), 3030, 2967, 2870, 1723, 1675, 1544, 1480, 1420, 1315 cm^−1^. GC/MS (70 eV): *m*/*z* (%) 215 (23) [M]^+^, 200 (100), 143 (8), 104 (10), 77 (20). HRMS (ESI): calcd. for C_13_H_18_N_3_ [M + H]^+^ 216.1501; found 216.1503.

## 4. Conclusions

In this paper we have described the first “green” and efficient condensation-mediated synthesis of 2-AIs successfully run in ChCl-based DESs as environmentally friendly, safe, and nonconventional reaction media. The scope of the reaction is broad in terms of the α-chloroketones employed, and both mono- and diphenyl-substituted guanidines proved to be competent partners. Compared to VOCs, shorter reaction times (4 to 6 h vs. 10 to 12 h) were required for the reaction to be completed in DESs, and the expected adducts could be isolated in very good yields (70%–80%), even with an operationally simple work-up procedure based on filtration and crystallization in the case of triaryl-substituted imidazoles prepared in a ChCl–urea eutectic mixture, which could be recycled. It is likely that some DES components (e.g., glycerol and urea) may speed up the reaction through a hydrogen bonding catalysis by activating both the carbonyl group and the guanidine derivative, thereby promoting first a nucleophilic substitution of the chloride ion, and subsequently the cyclodehydration reaction. Further investigation into the application of DESs both as reagents and catalysts in other condensation and multicomponent reactions are underway in our laboratory.

## Figures and Tables

**Table 1:** Synthesis of 2-aminoimidazoles **3** in a DES mixture or in other solvents ^a^. 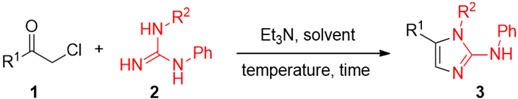

Entry	1	R^1^	2	R^2^	Solvent	Temperature	3, Yield (%) ^b^
1	**1a**	Ph	**2a**	Ph	DES	80	**3a**, 85
2	**1a**	Ph	**2a**	Ph	THF	reflux	**3a**, 81
3	**1b**	*t*-Bu	**2a**	Ph	DES	80	**3b**, 74
4	**1b**	*t*-Bu	**2a**	Ph	THF	reflux	**3b**, 78
5	**1c**	Me	**2a**	Ph	DES	80	**3c**, 70
6	**1c**	Me	**2a**	Ph	THF	reflux	**3c**, 71
7	**1d**	4-MeC_6_H_4_	**2a**	Ph	DES	80	**3d**, 80
8	**1d**	4-MeC_6_H_4_	**2a**	Ph	THF	reflux	**3d**, 78
9	**1e**	4-MeOC_6_H_4_	**2a**	Ph	DES	80	**3e**, 79
10	**1e**	4-MeOC_6_H_4_	**2a**	Ph	THF	reflux	**3e**, 77
11	**1f**	4-ClC_6_H_4_	**2a**	Ph	DES	80	**3f**, 80
12	**1f**	4-ClC_6_H_4_	**2a**	Ph	THF	reflux	**3f**, 83
13	**1g**	4-FC_6_H_4_	**2a**	Ph	DES	80	**3g**, 83
14	**1g**	4-FC_6_H_4_	**2a**	Ph	THF	reflux	**3g**, 85
15	**1a**	Ph	**2b** ^c^	H	DES	80	**3h**, 73
16	**1a**	Ph	**2b** ^c^	H	EtOH	reflux	**3h**, 76
17	**1b**	*t*-Bu	**2b** ^c^	H	DES	80	**3i**, 72
18	**1b**	*t*-Bu	**2b** ^c^	H	EtOH	reflux	**3i**, 70

^a^ Reaction conditions: α-chloroketone **1** (1.0 mmol), guanidine **2** (1.3 mmol), and Et_3_N (1.0 mmol); DES mixture: ChCl–Gly, 1:2 mol·mol^−1^, 2 mL per 1.0 mmol of **1**; reaction time: 4 to 6 h in DES mixtures, 10 to 12 h in THF, EtOH; ^b^ Isolated yield by column chromatography on silica gel; ^c^ See Materials and Methods for the preparation.

**Table 2:** Preparation of 2-aminoimidazole **3a,b,d**–**h** directly in a ChCl–urea (1:2) eutectic mixture ^a^. 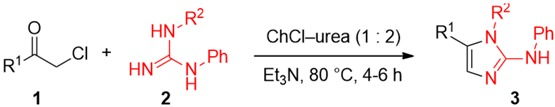

Entry	1	R^1^	2	R^2^	3, Yield (%)
1	**1a**	Ph	**2a**	Ph	**3a**, 75 ^b^
2	**1b**	*t*-Bu	**2a**	Ph	**3b**, 78 ^c^
3	**1d**	4-MeC_6_H_4_	**2a**	Ph	**3d**, 88 ^b^
4	**1e**	4-MeOC_6_H_4_	**2a**	Ph	**3e**, 84 ^b^
5	**1f**	4-ClC_6_H_4_	**2a**	Ph	**3f**, 76 ^b^
6	**1g**	4-FC_6_H_4_	**2a**	Ph	**3g**, 80 ^b^
7	**1a**	Ph	**2b**	H	**3h**, 65 ^c^

^a^ Reaction conditions: α-chloroketone **1** (1.0 mmol), guanidine **2** (1.3 mmol), and Et_3_N (1.0 mmol); DES mixture: ChCl–urea, 1:2 mol∙mol^−1^, 2 mL per 1.0 mmol of **1**; ^b^ Isolated yield by filtration from the crude reaction mixture and crystallization from Et_2_O/hexane; ^c^ Isolated yield by column chromatography on silica gel.

## Supplementary Materials

General procedures for the synthesis of 2-AIs in THF and EtOH and the ^1^H- and ^13^C-NMR spectra of compounds **3a**–**i** are available online at http://www.mdpi.com/1420-3049/21/7/924/s1.
