# Supplementary material for: An Expeditious and Greener Synthesis of 2-Aminoimidazoles in Deep Eutectic Solvents
Source: Molecules. 2016 Jul 16;21(7):924. doi: 10.3390/molecules21070924 (PMC6273198; doi:10.3390/molecules21070924)
Supplement: Supplementary file 1 [file molecules-21-00924-s001.pdf]

Supplementary Materials: An Expeditious and Greener Synthesis of 2-Aminoimidazoles in Deep Eutectic Solvents

Martina Capua, Serena Perrone, Filippo Maria Perna, Paola Vitale, Luigino Troisi[1], Antonio Salomone and Vito Capriati

1. General procedure for the synthesis of 2-aminoimidazoles **3a–g** in THF S2

2. General procedure for the synthesis of 2-aminoimidazoles **3h**,**i** in EtOH S2

3. Copies of the ^{1}H and ^{13}C-NMR spectra for compounds **3a–i** S3–S11

Synthesis of 2-Aminoimidazoles **3a**–**g** *in THF (Table 1, Main Manuscript)*

The appropriate α-chloroketone **1** (1.0 mmol), guanidine **2a** (1.3 mmol) and Et_3N (1 mmol) were added to dry THF (6 mL), under an argon atmosphere and magnetic stirring, and the mixture was then heated under reflux for a period of 10–12 h, until the ketone **1** disappeared, as revealed by GC-MS analysis. After this time, the mixture was cooled to room temperature and 10 mL of H_2O were added. The resulting aqueous solution was then extracted with AcOEt (3 × 10 mL). The combined organic phases were dried over Na_2SO_4 and concentrated in vacuo. The crude product was purified by flash-chromatography (silica gel; petroleum ether/AcOEt 80:20–95:5) to give products **3a**–**g**.

Synthesis of 2-Aminoimidazoles **3h**,**i** *in EtOH (Table 1, Main Manuscript)*

Guanidinium carbonate **2b**·H_2CO_3 (1.3 mmol) and KOH (1.3 mmol) were added to EtOH (6 mL) under magnetic stirring, and the mixture was then heated under reflux for a period of 30 min, so as to liberate the free base of guanidine **2b** in situ. After this time, α-chloroketone **1** and triethylamine (1.3 mmol) were added and the reaction stirred at reflux for 10–12 h until the ketone **1** disappeared, as revealed by GC-MS analysis. The reaction mixture was then cooled to room temperature and 10 mL of H_2O were added. The resulting aqueous solution was extracted with AcOEt (3 × 10 mL), and the combined organic phases were dried over Na_2SO_4 and concentrated in vacuo. The crude product was purified by flash-chromatography (silica gel; petroleum ether/AcOEt 20:80–40:60) to give products **3h**–**i**.

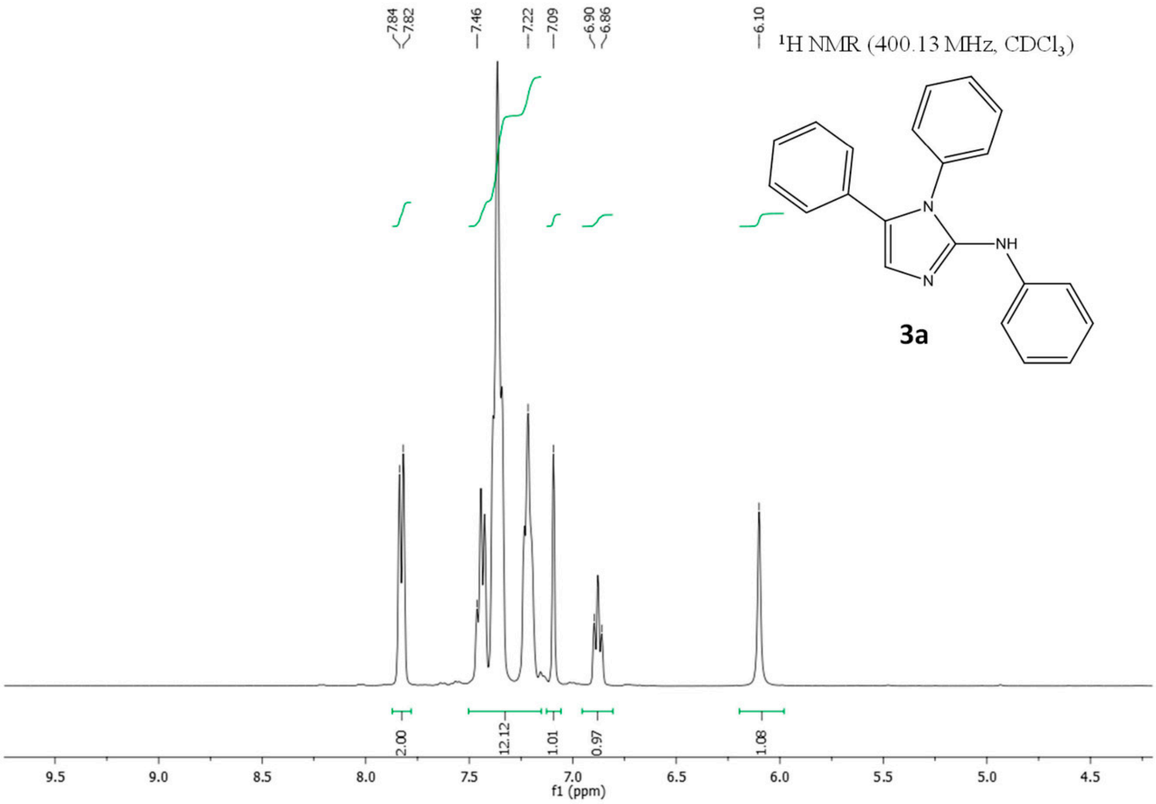
7.84
7.82
7.46
7.22
7.09
6.90
6.86
6.10
¹H NMR (400.13 MHz, CDCl3)
N
NH
N
3a
2.00
12.12
1.01
0.97
1.08
9.5
9.0
8.5
8.0
7.5
7.0
6.5
6.0
5.5
5.0
4.5
f1 (ppm)

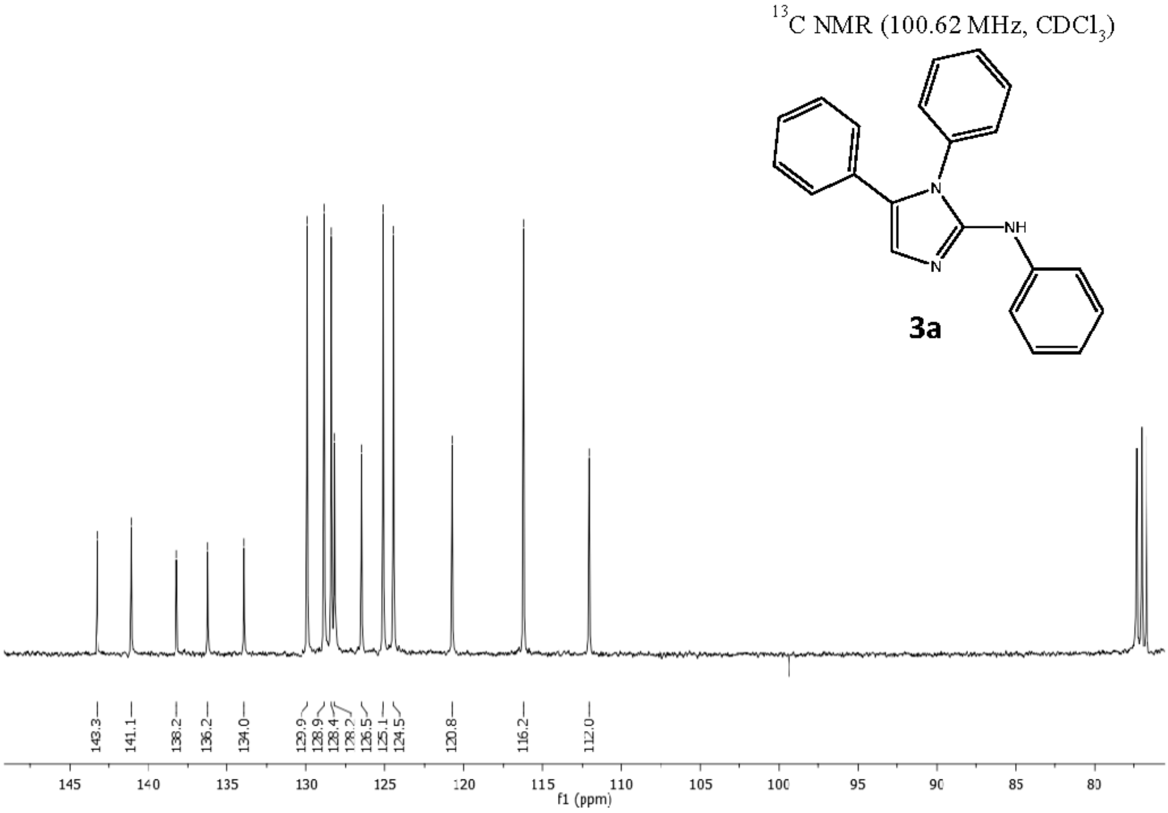
13C NMR (100.62 MHz, CDCl3)
N
NH
N
3a
143.3
141.1
138.2
136.2
134.0
129.9
128.9
128.4
128.2
126.5
125.1
124.5
120.8
116.2
112.0
145
140
135
130
125
120
115
110
105
100
95
90
85
80
f1 (ppm)

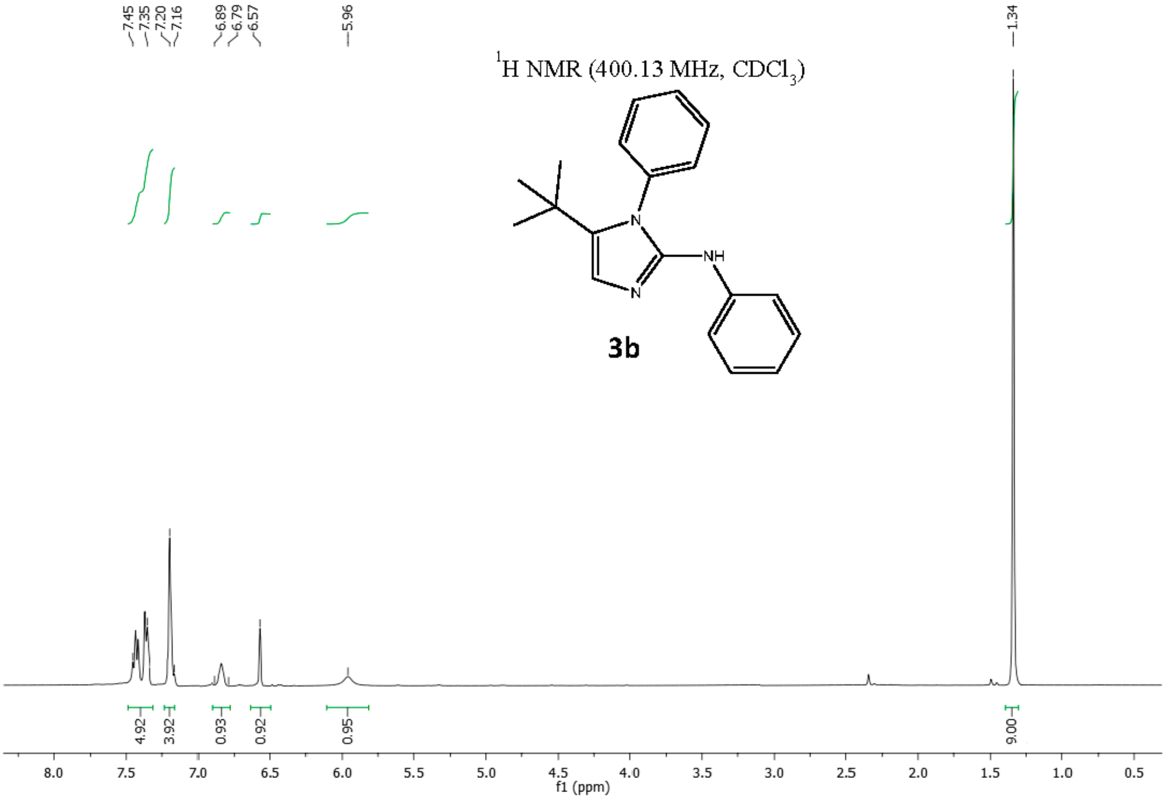

7.45
7.35
7.20
7.16
6.89
6.79
6.57
5.96
1.34
1H NMR (400.13 MHz, CDCl3)
N
NH
N
3b
4.92
3.92
0.93
0.92
0.95
9.00
8.0 7.5 7.0 6.5 6.0 5.5 5.0 4.5 4.0 3.5 3.0 2.5 2.0 1.5 1.0 0.5
f1 (ppm)

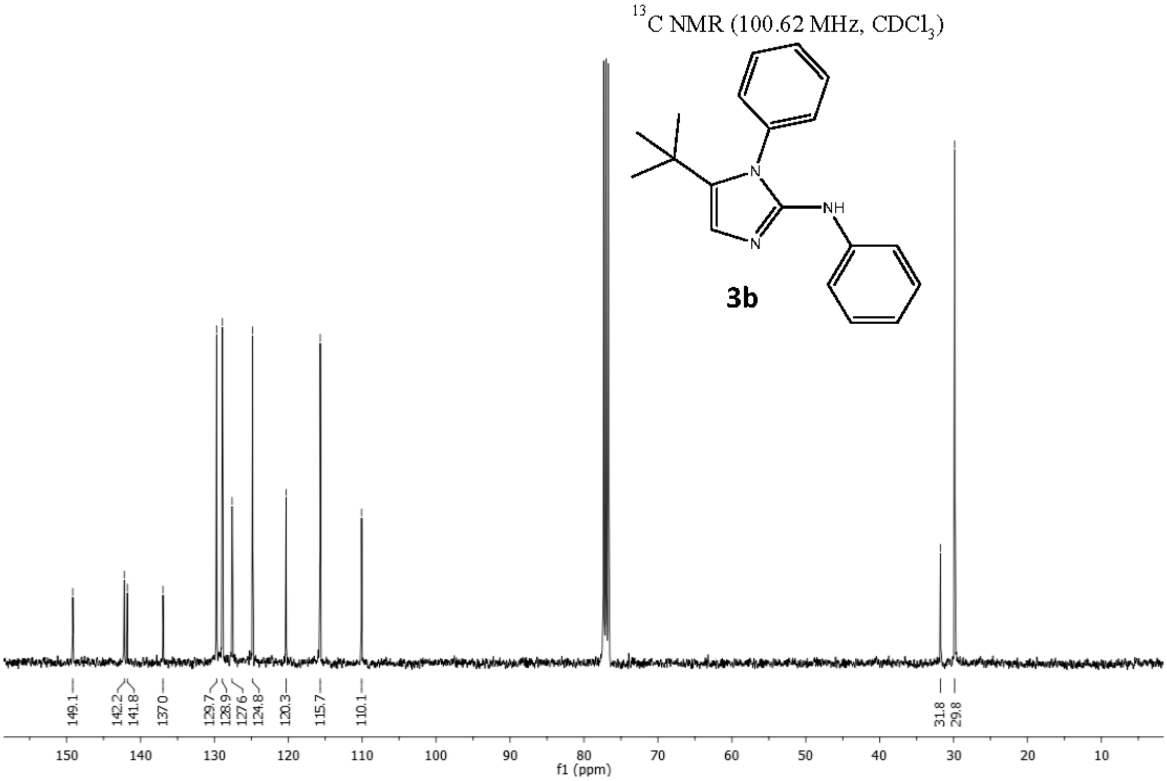

13C NMR (100.62 MHz, CDCl3)
N
NH
N
3b
149.1
142.2
141.8
137.0
129.7
128.9
127.6
124.8
120.3
115.7
110.1
31.8
29.8
150 140 130 120 110 100 90 80 70 60 50 40 30 20 10
f1 (ppm)

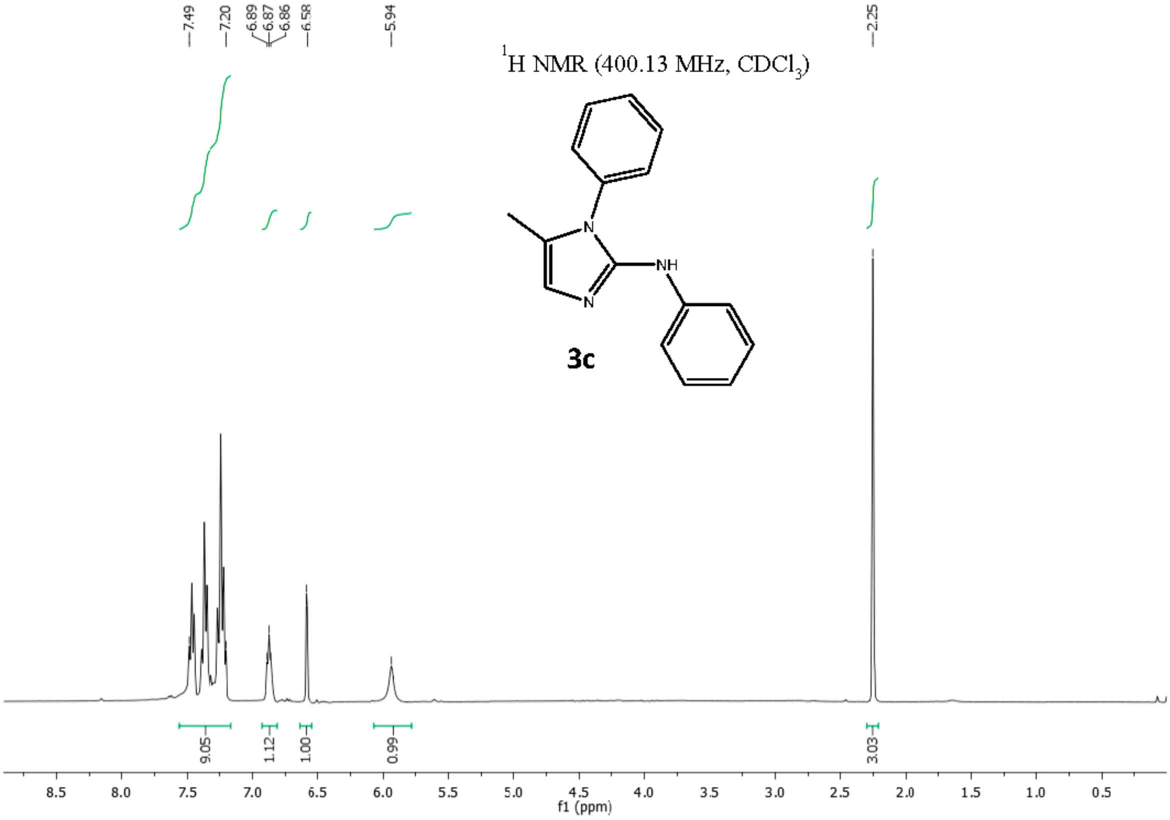

7.49
7.20
6.89
6.87
6.86
6.58
5.94
2.25
1H NMR (400.13 MHz, CDCl3)
N
NH
N
3c
9.05
1.12
1.00
0.99
3.03
8.5 8.0 7.5 7.0 6.5 6.0 5.5 5.0 4.5 4.0 3.5 3.0 2.5 2.0 1.5 1.0 0.5
f1 (ppm)

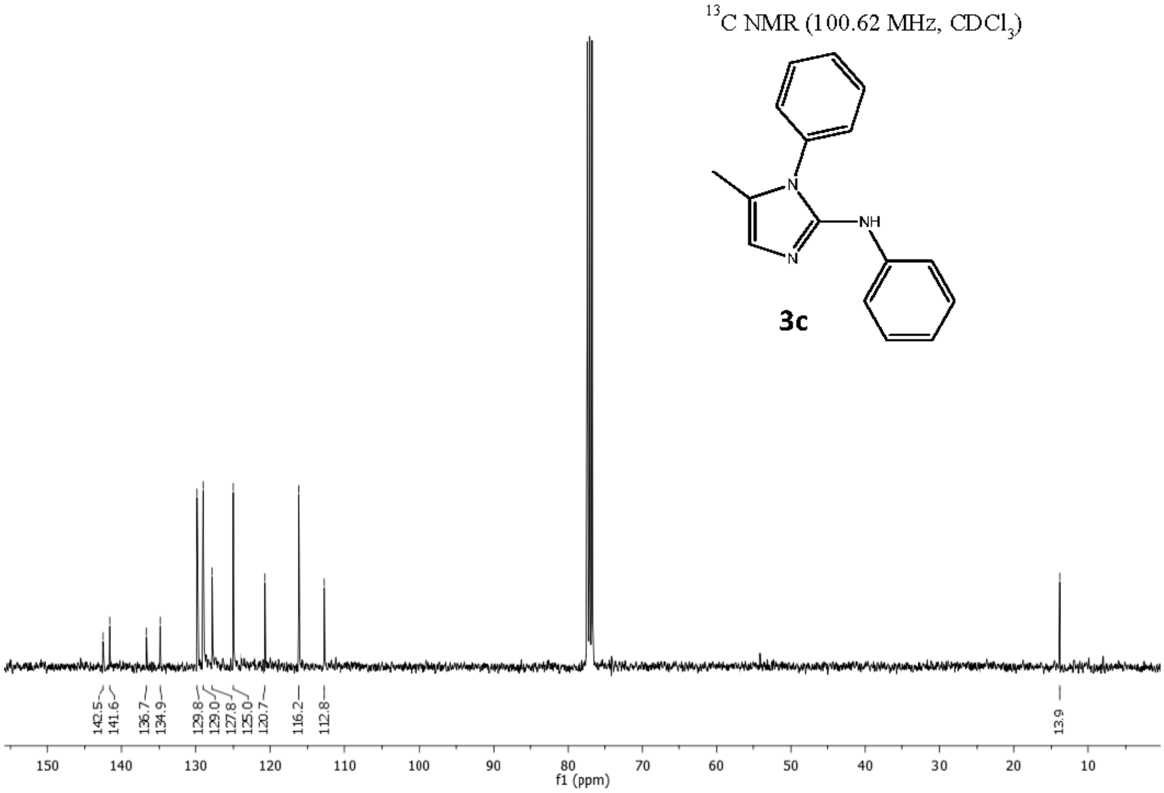

13C NMR (100.62 MHz, CDCl3)
N
NH
N
3c
142.5
141.6
136.7
134.9
129.8
129.0
127.8
125.0
120.7
116.2
112.8
13.9
150 140 130 120 110 100 90 80 70 60 50 40 30 20 10
f1 (ppm)

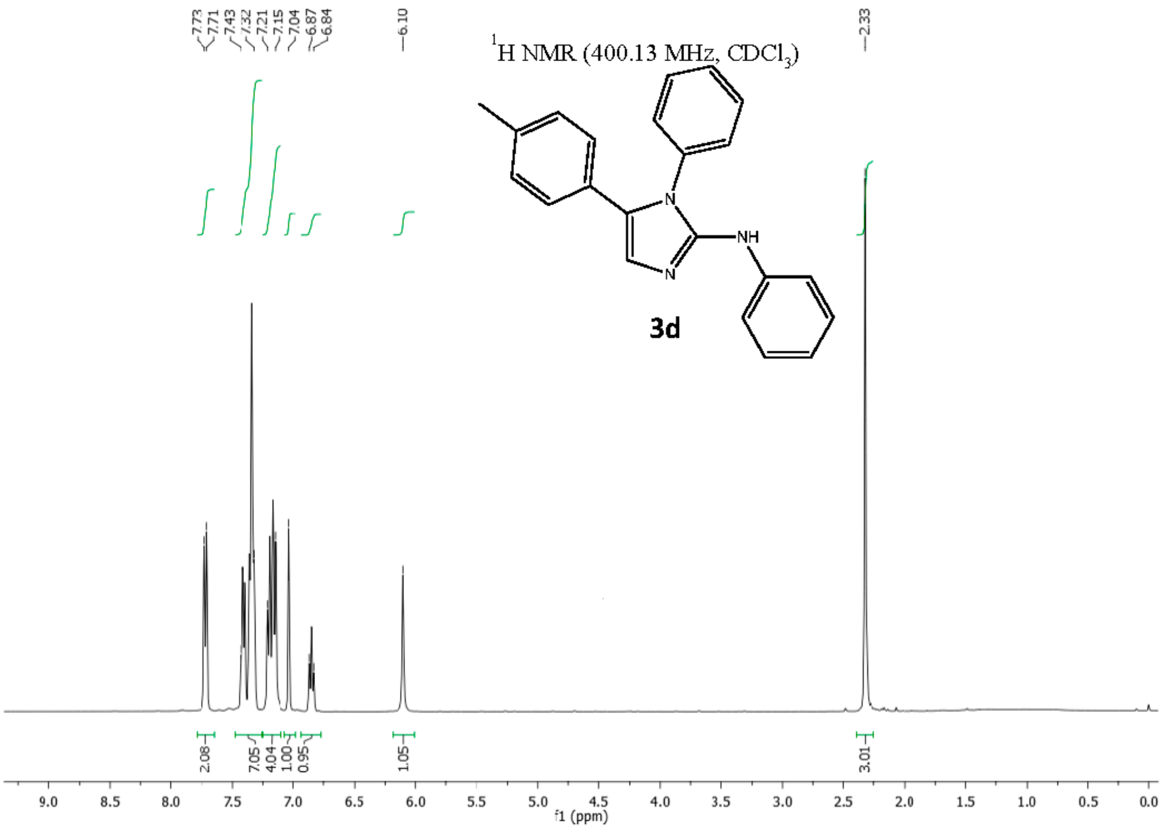

7.73
7.71
7.43
7.32
7.21
7.15
7.04
6.87
6.84
6.10
2.33
^{1}H NMR (400.13 MHz, CDCl3)
N
NH
N
3d
2.08
7.05
4.04
1.00
0.95
1.05
3.01
9.0
8.5
8.0
7.5
7.0
6.5
6.0
5.5
5.0
4.5
4.0
3.5
3.0
2.5
2.0
1.5
1.0
0.5
0.0
f1 (ppm)

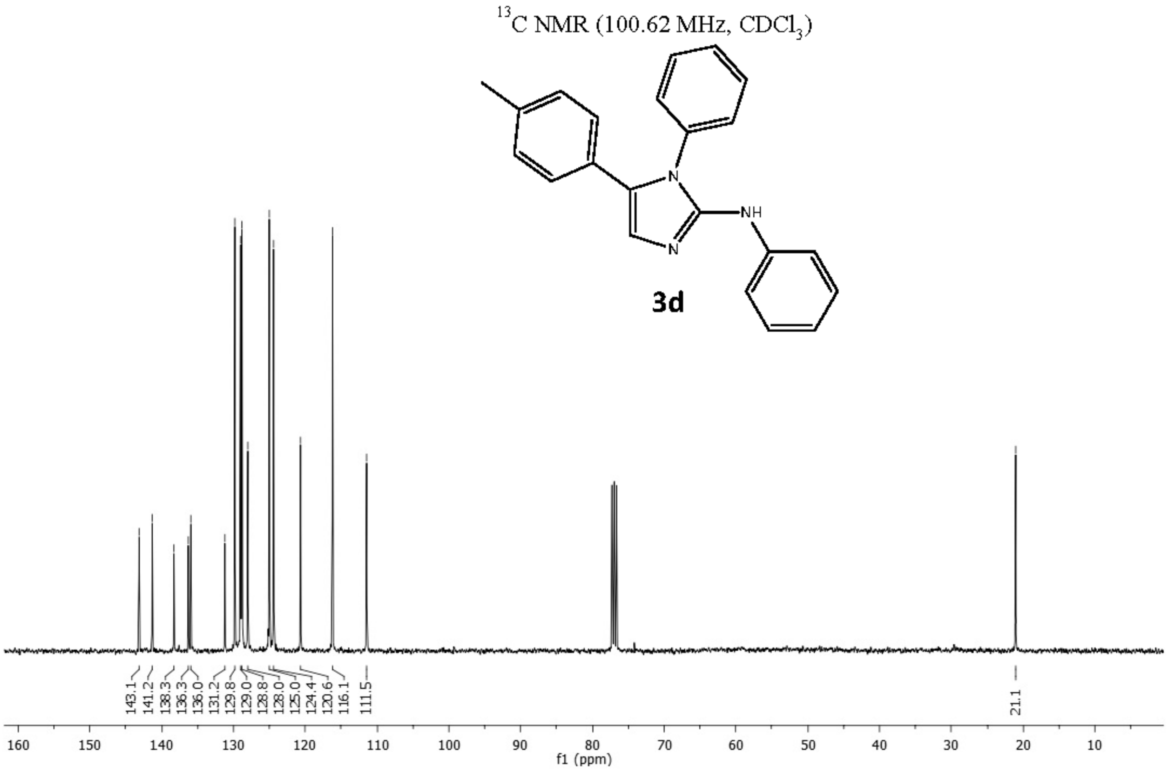

^{13}C NMR (100.62 MHz, CDCl3)
N
NH
N
3d
143.1
141.2
138.3
136.3
136.0
131.2
129.8
129.0
128.8
128.0
125.0
124.4
120.6
116.1
111.5
21.1
160
150
140
130
120
110
100
90
80
70
60
50
40
30
20
10
f1 (ppm)

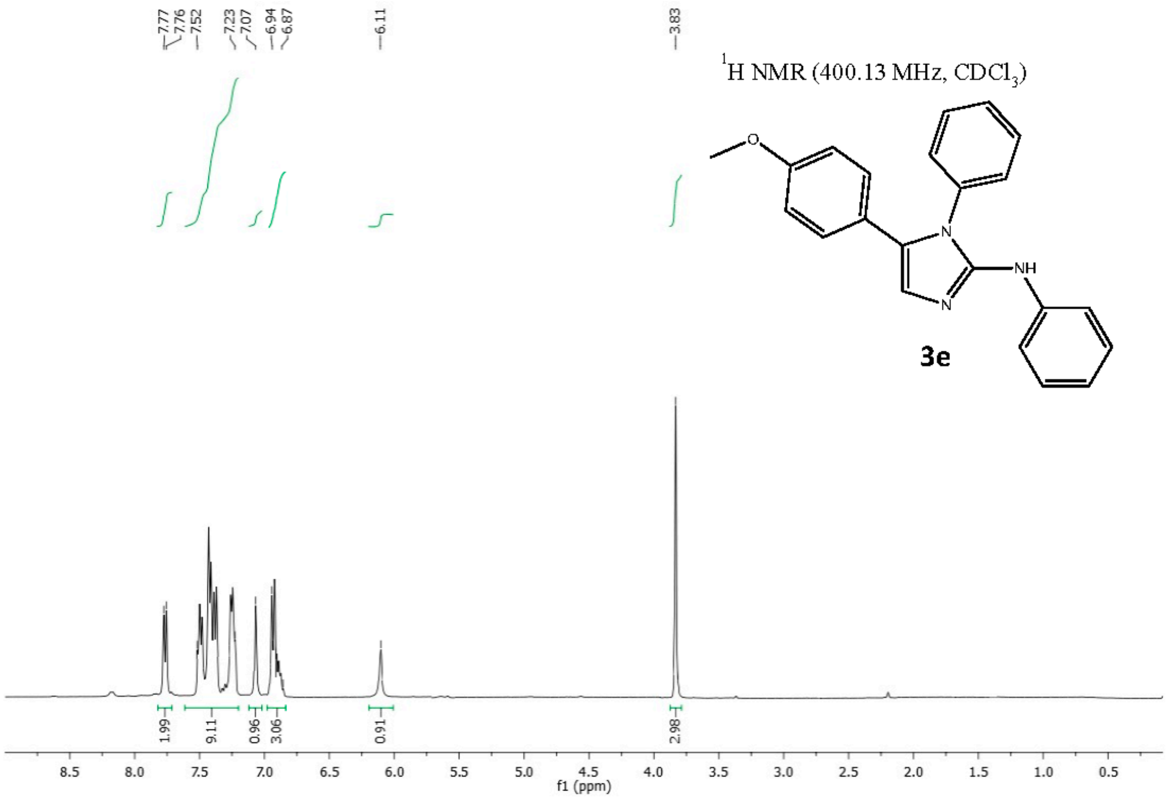
7.77
7.76
7.52
7.23
7.07
6.94
6.87
6.11
3.83
^{1}H NMR (400.13 MHz, $CDCl_3$)
O
N
NH
N
3e
1.99
9.11
0.96
3.06
0.91
2.98
8.5
8.0
7.5
7.0
6.5
6.0
5.5
5.0
4.5
4.0
3.5
3.0
2.5
2.0
1.5
1.0
0.5
f1 (ppm)

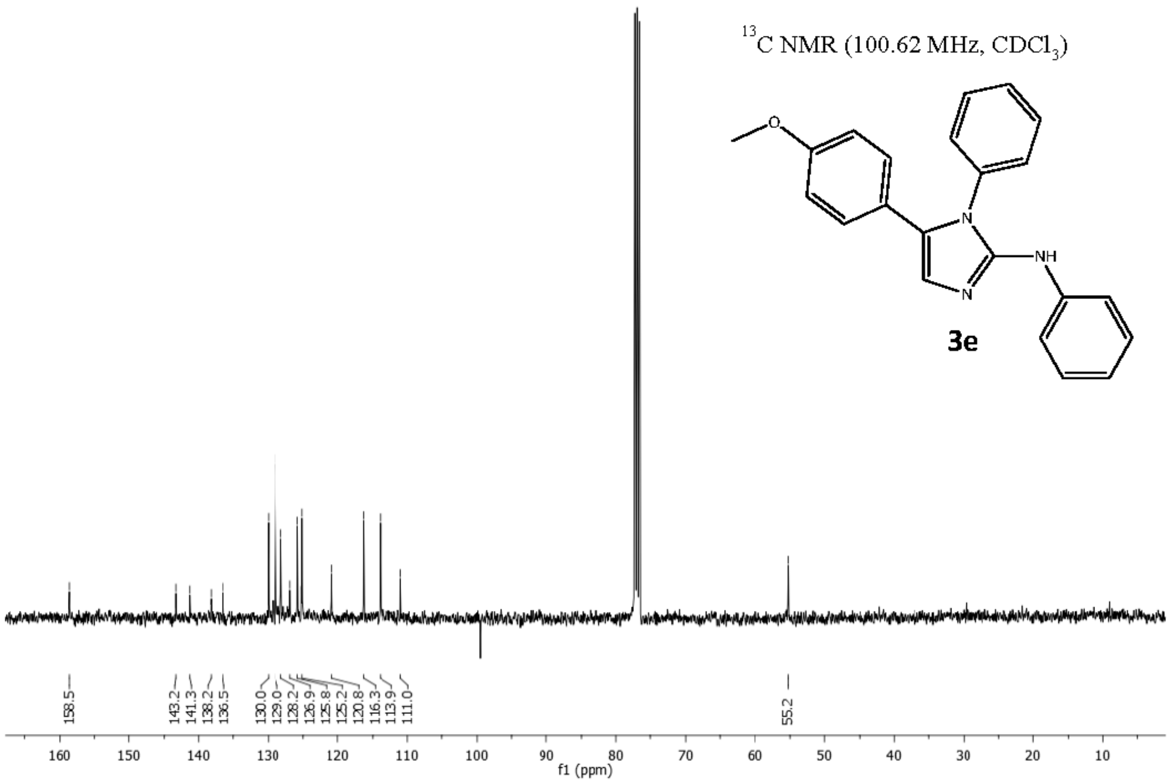
^{13}C NMR (100.62 MHz, $CDCl_3$)
O
N
NH
N
3e
158.5
143.2
141.3
138.2
136.5
130.0
129.0
128.2
126.9
125.8
125.2
120.8
116.3
113.9
111.0
55.2
160
150
140
130
120
110
100
90
80
70
60
50
40
30
20
10
f1 (ppm)

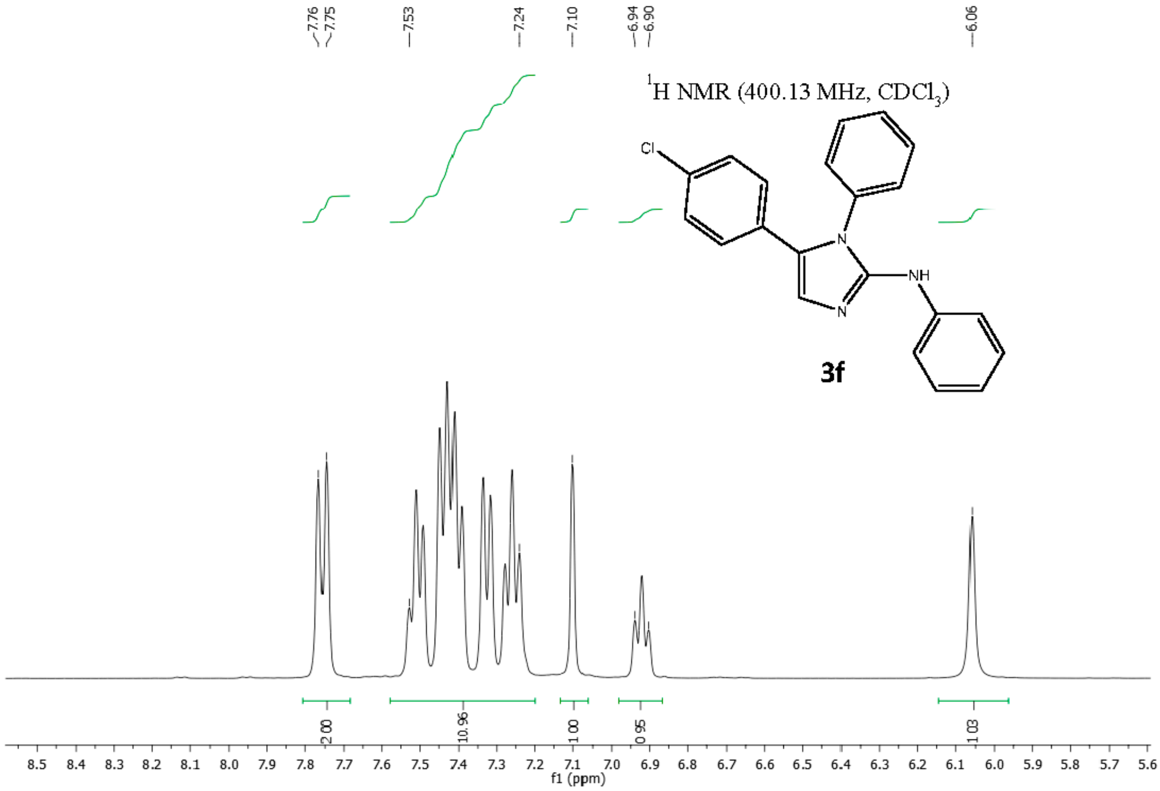

7.76
7.75
7.53
7.24
7.10
6.94
6.90
6.06
1H NMR (400.13 MHz, CDCl3)
Cl
N
NH
N
3f
2.00
10.96
1.00
0.95
1.03
8.5 8.4 8.3 8.2 8.1 8.0 7.9 7.8 7.7 7.6 7.5 7.4 7.3 7.2 7.1 7.0 6.9 6.8 6.7 6.6 6.5 6.4 6.3 6.2 6.1 6.0 5.9 5.8 5.7 5.6
f1 (ppm)

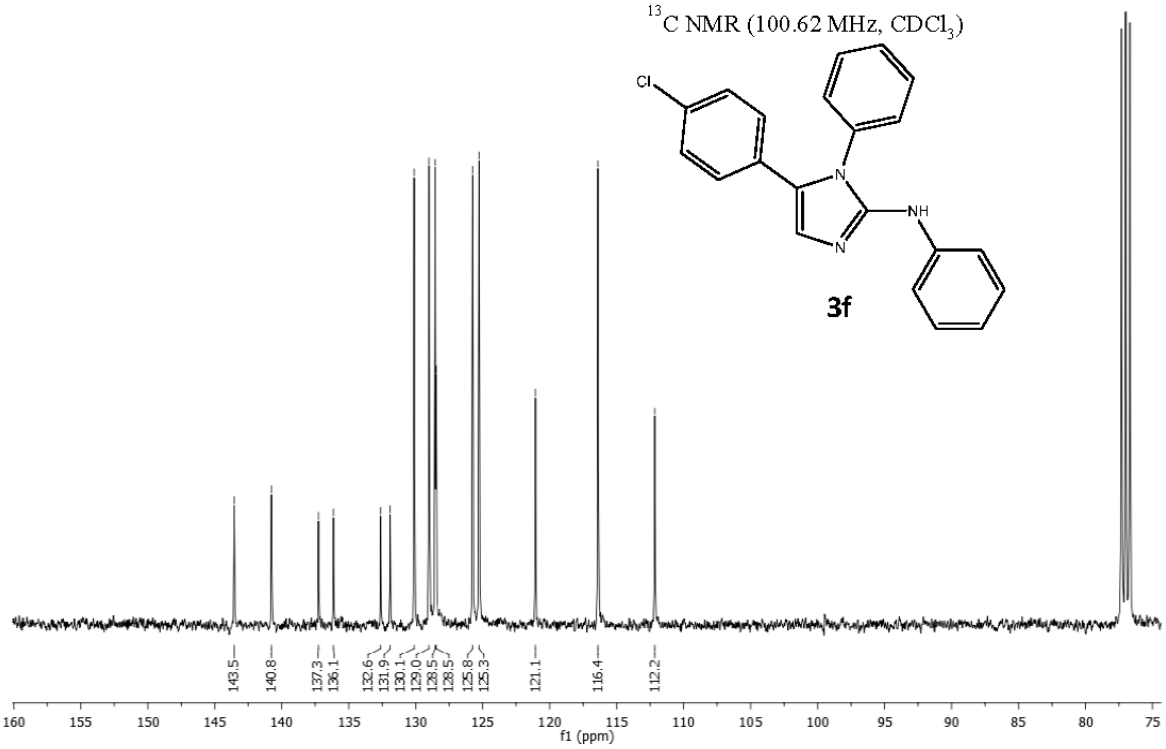

13C NMR (100.62 MHz, CDCl3)
Cl
N
NH
N
3f
143.5
140.8
137.3
136.1
132.6
131.9
130.1
129.0
128.5
128.5
125.8
125.3
121.1
116.4
112.2
160 155 150 145 140 135 130 125 120 115 110 105 100 95 90 85 80 75
f1 (ppm)

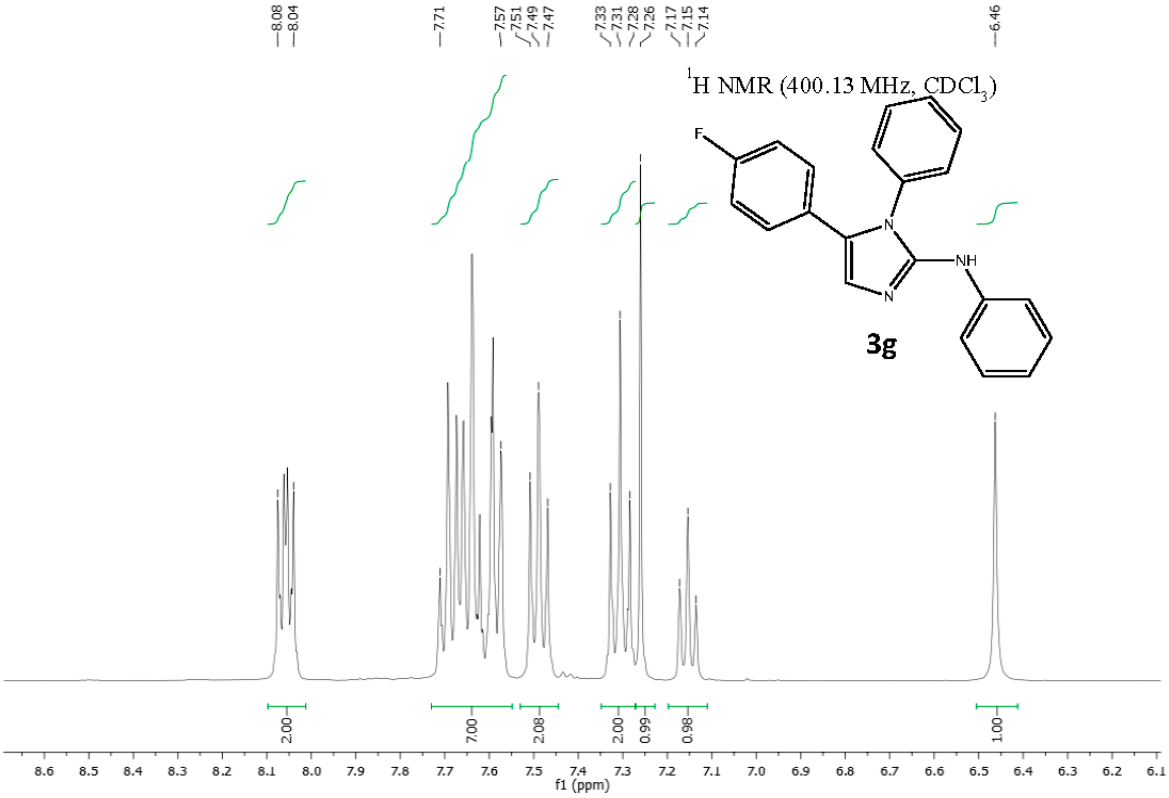

^{13}C NMR (100.62 MHz, $CDCl_3$)

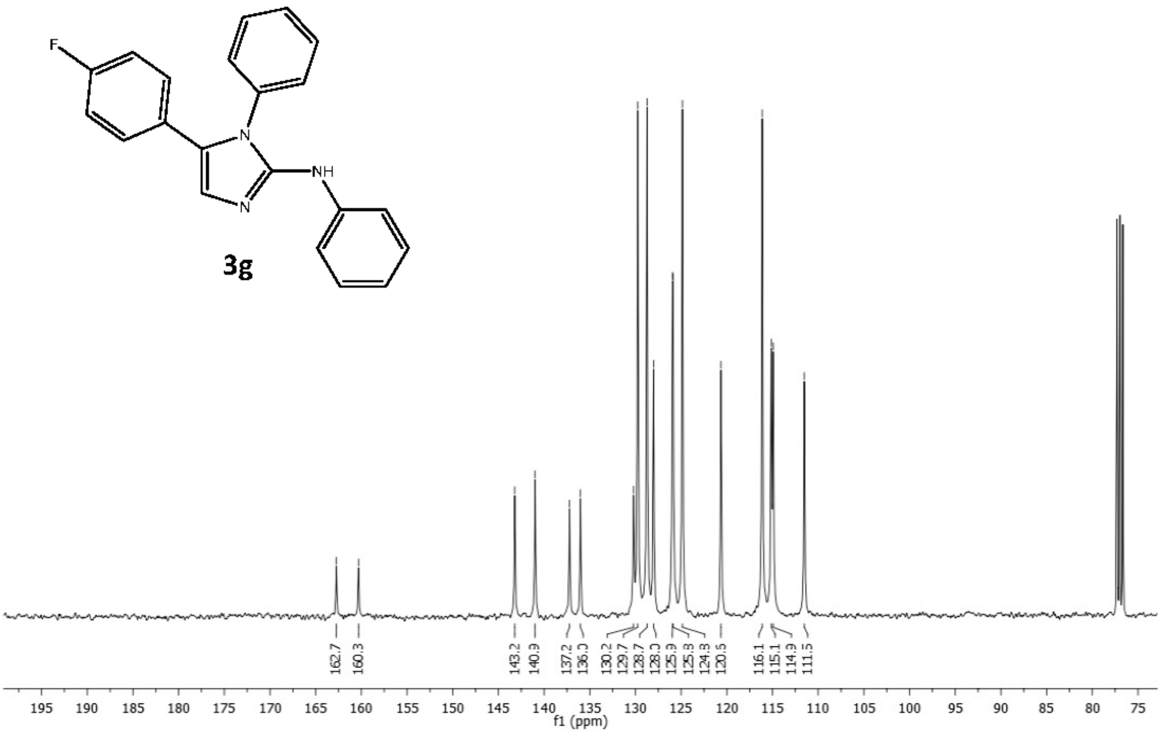

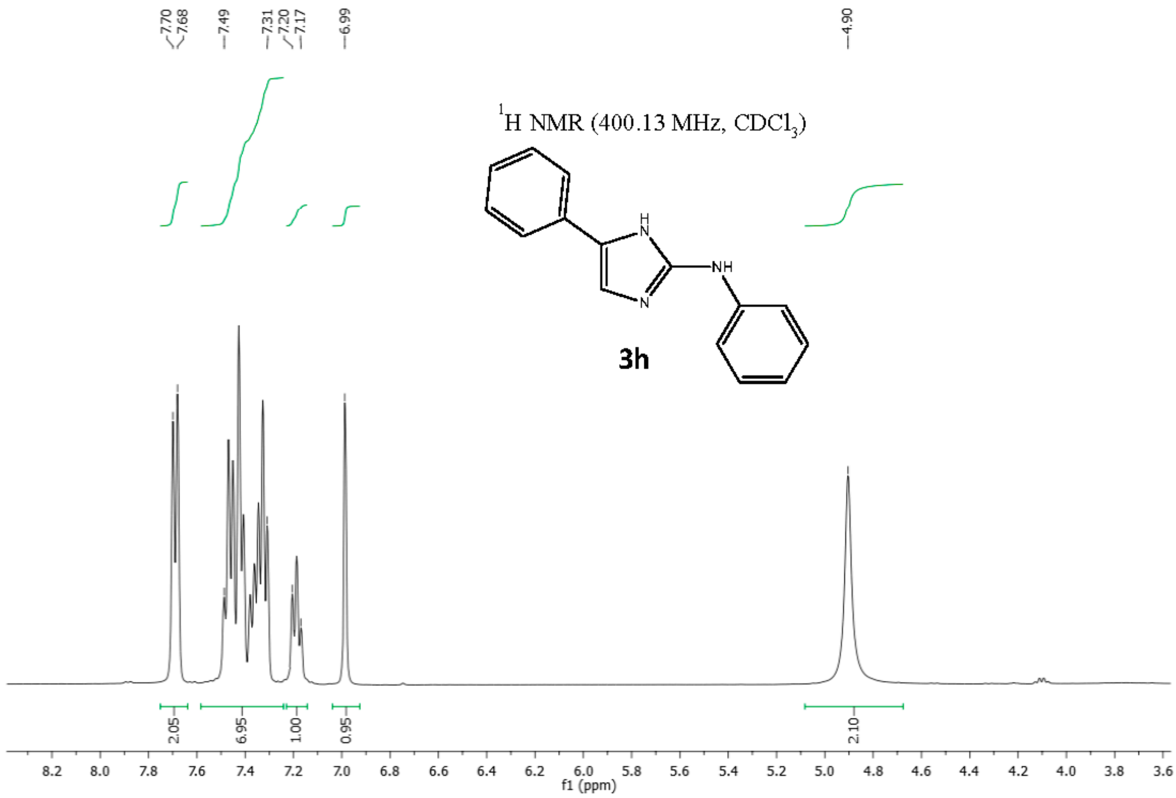

7.70
7.68
7.49
7.31
7.20
7.17
6.99
4.90
1H NMR (400.13 MHz, CDCl3)
H
N
NH
N
3h
2.05
6.95
1.00
0.95
2.10
8.2
8.0
7.8
7.6
7.4
7.2
7.0
6.8
6.6
6.4
6.2
6.0
5.8
5.6
5.4
5.2
5.0
4.8
4.6
4.4
4.2
4.0
3.8
3.6
f1 (ppm)

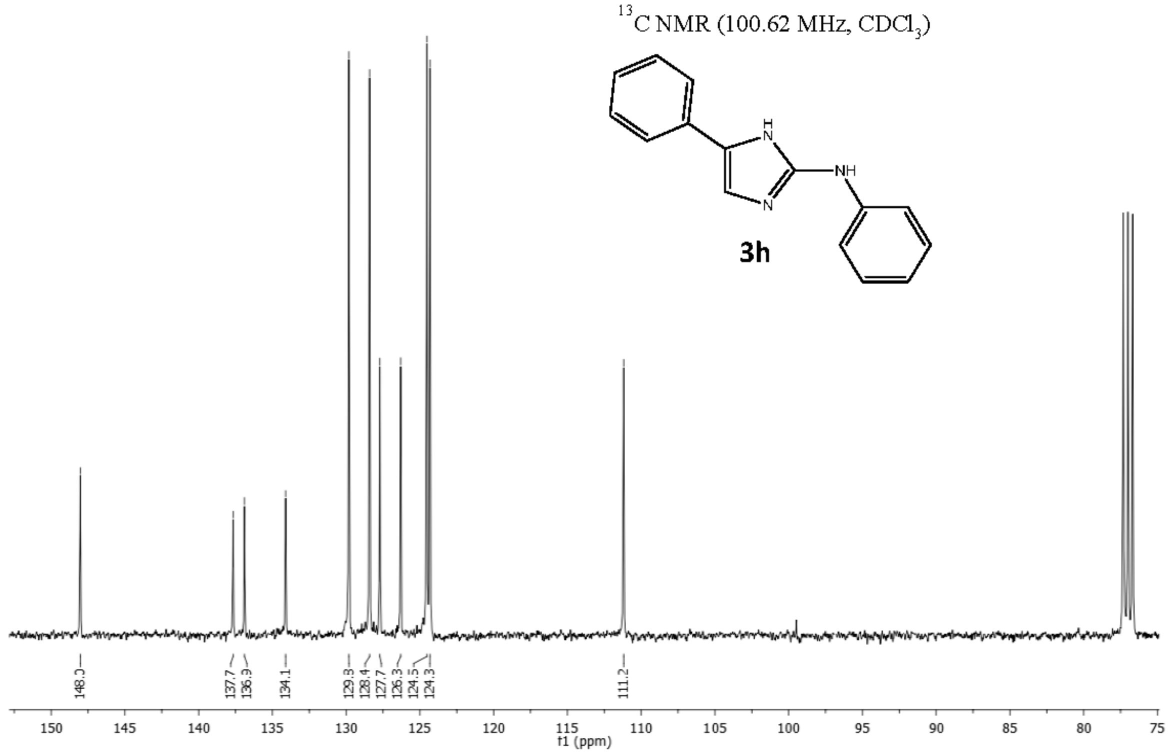

13C NMR (100.62 MHz, CDCl3)
H
N
NH
N
3h
148.0
137.7
136.9
134.1
129.8
128.4
127.7
126.3
124.5
124.3
111.2
150
145
140
135
130
125
120
115
110
105
100
95
90
85
80
75
f1 (ppm)

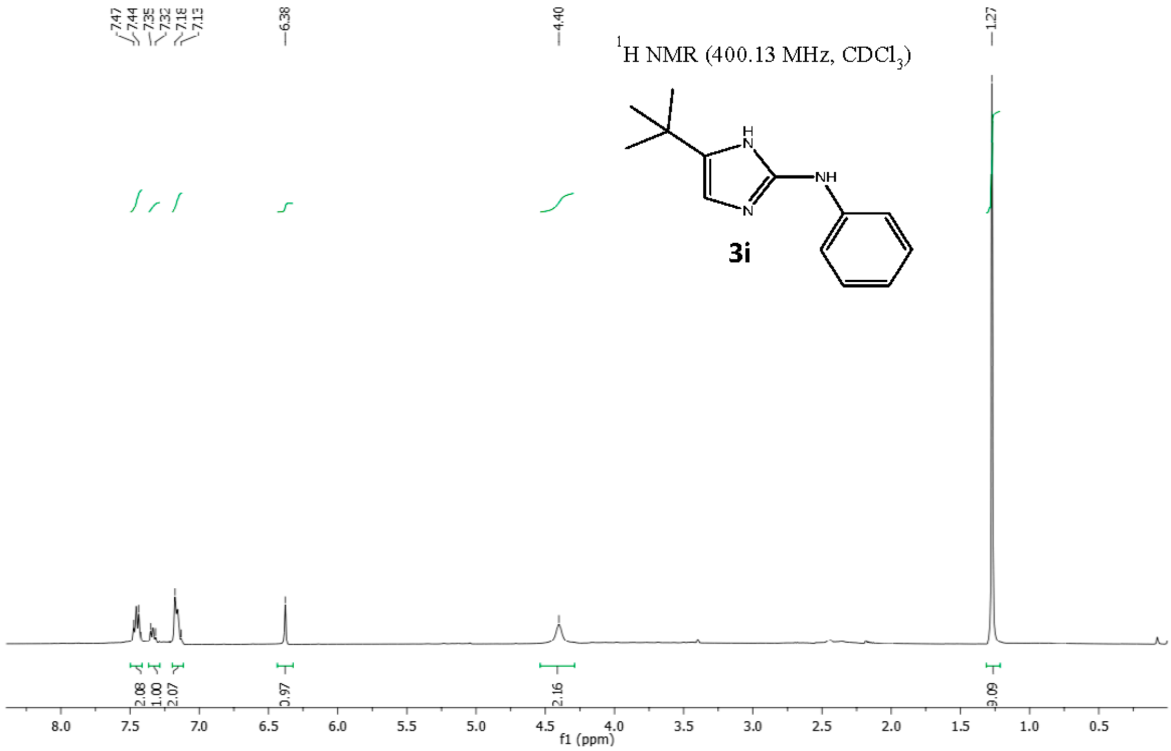
^{1}H NMR (400.13 MHz, $CDCl_3$)
3i
NH
H
N
N
7.47
7.44
7.35
7.32
7.18
7.13
6.38
4.40
1.27
2.08
1.00
2.07
0.97
2.16
9.09
8.0 7.5 7.0 6.5 6.0 5.5 5.0 4.5 4.0 3.5 3.0 2.5 2.0 1.5 1.0 0.5
f1 (ppm)

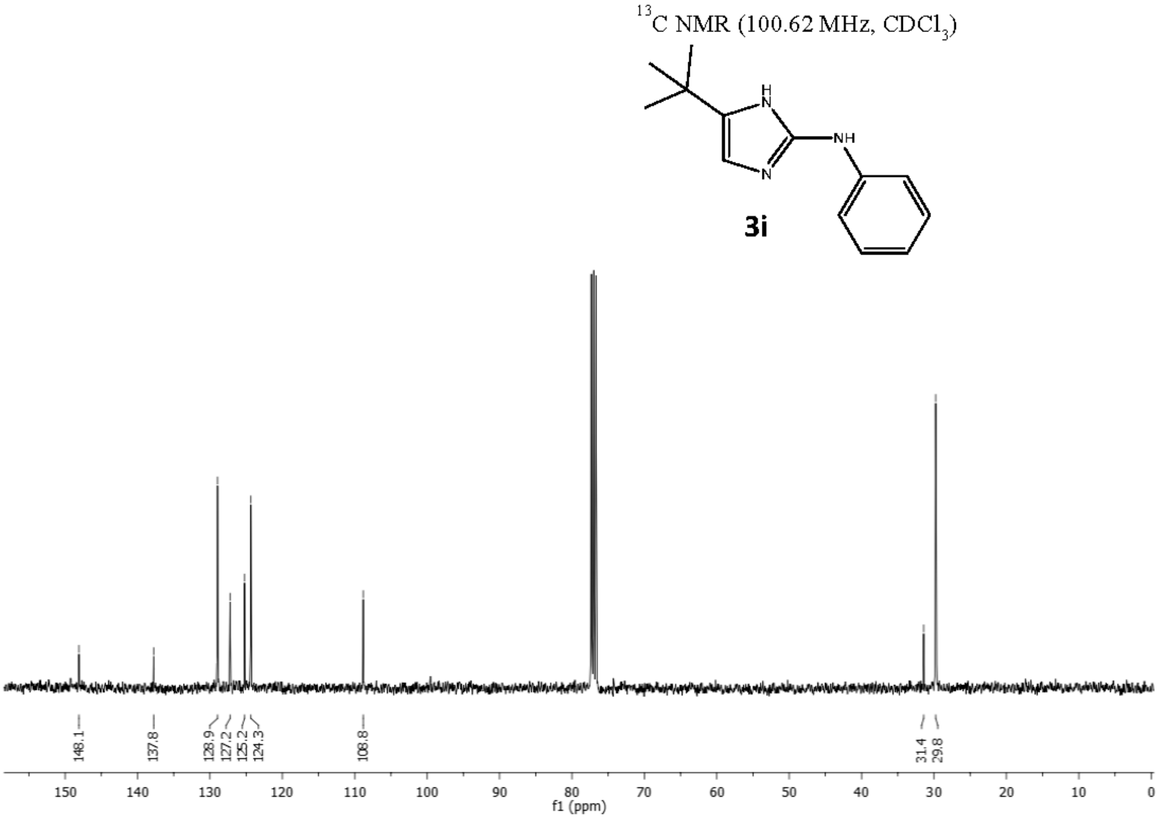
^{13}C NMR (100.62 MHz, $CDCl_3$)
3i
NH
H
N
N
148.1
137.8
128.9
127.2
125.2
124.3
108.8
31.4
29.8
150 140 130 120 110 100 90 80 70 60 50 40 30 20 10 0
f1 (ppm)
